# Natural Variation in an ABC Transporter Gene Associated with Seed Size Evolution in Tomato Species

**DOI:** 10.1371/journal.pgen.1000347

**Published:** 2009-01-23

**Authors:** Cintia Hotta Orsi, Steven D. Tanksley

**Affiliations:** 1 Department of Plant Breeding and Genetics, Cornell University, Ithaca, New York, United States of America; 2 Department of Plant Biology, Cornell University, Ithaca, New York, United States of America; University of Chicago, United States of America

## Abstract

Seed size is a key determinant of evolutionary fitness in plants and is a trait that often undergoes tremendous changes during crop domestication. Seed size is most often quantitatively inherited, and it has been shown that *Sw4.1* is one of the most significant quantitative trait loci (QTLs) underlying the evolution of seed size in the genus *Solanum*—especially in species related to the cultivated tomato. Using a combination of genetic, developmental, molecular, and transgenic techniques, we have pinpointed the cause of the *Sw4.1* QTL to a gene encoding an ABC transporter gene. This gene exerts its control on seed size, not through the maternal plant, but rather via gene expression in the developing zygote. Phenotypic effects of allelic variation at *Sw4.1* are manifested early in seed development at stages corresponding to the rapid deposition of starch and lipids into the endospermic cells. Through synteny, we have identified the Arabidopsis *Sw4.1* ortholog. Mutagenesis has revealed that this ortholog is associated with seed length variation and fatty acid deposition in seeds, raising the possibility that the ABC transporter may modulate seed size variation in other species. Transcription studies show that the ABC transporter gene is expressed not only in seeds, but also in other tissues (leaves and roots) and, thus, may perform functions in parts of the plants other than developing seeds. Cloning and characterization of the *Sw4.1* QTL gives new insight into how plants change seed during evolution and may open future opportunities for modulating seed size in crop plants for human purposes.

## Author Summary

Given fixed resources, plants have a choice whether to produce many small seeds or a few large seeds. In terms of reproductive fitness, there are costs and benefits to both strategies. As a result, plant species vary more than 100,000-fold in both seed size and seed output. The current study focuses on understanding the molecular and developmental basis of a single genetic locus (or quantitative trait locus) that determines seed size between the cultivated tomato and its wild relatives. We show that the cause of size variation can be traced to a gene encoding an ABC transporter protein. The gene apparently exercises its control on seed size through expression in the developing seeds and not the mother plant that nurtures those seeds. A comparison with the model plant *Arabidopsis thaliana* suggests that the ABC transporter identified in tomato may also control seed size in other plants, opening research opportunities for understanding plant adaptation and for potentially modulating seed size in crop plants for human purposes.

## Introduction

**Figure 1:**
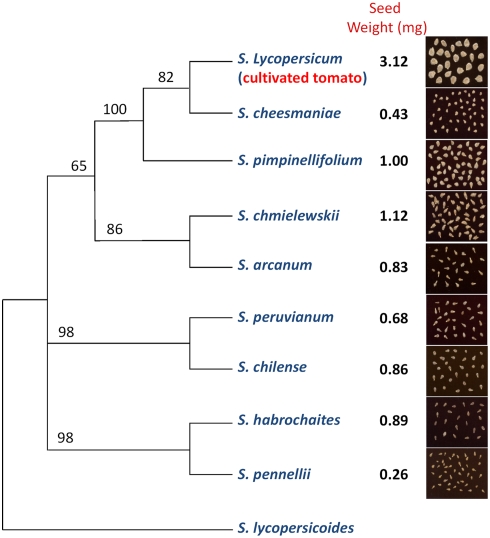
Phylogenetic relationships of species in the genus *Solanum* most closely related to the cultivated tomato. Numbers at nodes indicates bootstrap values. Modified from Spooner et al. [55] and Peralta et al. [56].

Seeds represent the vehicle by which plants vie for evolutionary success. A key feature of seeds is their size, which in turn is one of the most variable traits in the plant kingdom. Seeds range in weight from less than 1 microgram in the Coral-root orchid (*Corallorhiza maculate*) to more than 10 kg in the Coco-de-mer palm (*Lodoicea maldivica*). This large range can be observed not only among taxa, but also within taxa. For example, contained in the genus *Solanum* are a set of 9 cross compatible species closely related to the tomato. Despite their close taxonomic affinities, these species show a 10-fold range in seed size suggesting a rapid rate of evolutionary change (Figure 1).

Why plants vary so much in seed size is not known. However, evolutionists and ecologists have long noted this great variation and hypothesized its importance in adaptation. In terms of survival, there are both risks and benefits for a species to increase (or decrease) seed size. Because maternal resources are limited, a species has to “decide” whether to invest energy into a few large seeds or many small seeds [1],[2]. Intra- and interspecific studies of offspring fitness in plant communities have demonstrated that plants producing a small number of large seeds often have higher tolerance to drought [3], herbivory [4], shading [5], and nutrient-deficient soils [6]. However, plants producing a large number of small seeds exhibit superior colonization abilities with the advantage of dispersal due to the abundance of seeds and higher likelihood to escape from predation [7],[8].

Scientific interest in seed size relates not only to its importance in evolution, but also to crop domestication. Crops domesticated for consumption of their seeds (e.g. soybean, wheat, sunflower) often produce seeds significantly larger than their wild ancestors [9]–[12]. It is likely that early humans consciously selected for larger seeds in an effort to increase yield and improve harvest efficiency. However, seed size also increased during domestication in crops not harvested for their edible seed. For example, domesticated tomatoes produce seeds up to several fold larger than their wild ancestors (Figure 1) [13]. Likewise cultivated squash (*Cucurbita pepo*) produce seed more than two fold larger than their wild counter parts [14]. Why seed size increased during domestication in crops not consumed for their seeds is unclear. However, it has been conjectured that seed size increased in these species due to indirect selection for greater seedling vigor and germination uniformity under field production [15].

Despite the importance of seed size in plant evolution and crop domestication, relatively little is known about the genetic and molecular processes underlying natural variation in seed size. Most of our knowledge comes from quantitative trait mapping studies which have revealed a fairly large number of QTL affecting seed size in a variety of plants – e.g. Arabidopsis [16], rice [17]–[22], soybean [23],[24], sunflower [11], [25]–[28]. However, most of these studies have not gone beyond the mapping stage and hence provide little insight into the developmental and molecular mechanisms underpinning seed size variation. The exception is rice where three seed size QTLs have been recently cloned. These encode a previously unknown RING-type E3 ubiquitin ligase [29], a putative transmembrane protein [30], and deletion of a gene of unknown function [17].

Tomato is one of the few species not domesticated for edible seeds, where extensive QTL mapping for seed size has been conducted. Over the past 25 years, mapping studies involving crosses between the cultivated tomato and related wild species have revealed approximately 20 QTLs which account for most seed size variation [13], [31]–[34]. Different subsets of these QTLs were identified in different studies. However, a common feature of all studies was that a major QTL on chromosome 4 (referred to as *Seed weight 4.1* or *Sw4.1*) invariably accounted for a large portion of the genetic variation for seed size. *Sw4.1* is responsible for up to 25% of the total phenotypic variation in segregating populations and up to 54% of the seed weight variation in crosses between nearly isogenic lines [13]. The conservation of *Sw4.1* across tomato species, and its potential role in the evolution and domestication of cultivated tomato, makes *Sw4.1* a prime candidate for characterization and cloning. Thus the objective of this study was to uncover the genetic, developmental and molecular mechanisms underlying modulation of seed size by the *Sw4.1* QTL.

## Results/Discussion

### *Sw4.1* Controls Seed Weight through Zygotic Effects

The size or weight of a seed can potentially be affected by the genotype of three different plant parts/tissues: a) the female plant bearing the fruit which contains the developing seed and contributes the testa; b) the triploid endosperm which nourishes the developing embryo and c) the diploid embryo. A maternal effect would be caused by a substantial contribution of the maternal genotype from the testa (a) and/or endosperm (b) to the seed development, while a zygotic effect would be attributed to the equal contribution of maternal and paternal genotypes from the zygote (c).

To differentiate maternal from zygotic effects, a reciprocal cross experiment was conducted using a pair of nearly isogenic lines (NILs) (see Material and Methods). In Cross 1, an inbred line homozygous for a “large-seed” (*L/L*) allele from *S. lycopersicum* was used as the female in a cross with a nearly isogenic line (NIL) homozygous for the “small-seed” (*S/S*) allele from *S. pimpinellifolium*. In Cross 2, the reciprocal cross was performed using the *S/S* as the female parent. For Cross 1, the F1 seed would develop on a maternal plant of the *L/L* genotype, whereas with Cross 2, the seed would develop on a maternal plant of the *S/S* genotype. If *Sw4.1* exerts its effect on seed weight through the maternal parent, F1 seed from Cross 1 should be significantly larger than F1 seed from Cross 2. The results from these experiments revealed that reciprocal crosses result in seed indistinguishable in weight: 3.07 mg (Cross 1) versus 3.10 mg (Cross 2) (*P* = 0.99). Whereas self-pollination of the same parents, *L/L* and *S/S*, resulted in seeds weighting 3.34 mg and 2.63 mg respectively (*P*<0.005).

The equivalency in seed weight for the reciprocal F1s suggests that *Sw4.1* does not influence seed weight through any significant effect exerted by the genotype of the maternal environment (i.e., fruit). It should be noted that reciprocal crosses would result in triploid endosperm with different parental allelic dosage (*L/L/S* versus *L/S/S*). Such differences in allelic dosage might also cause differences in seed weight between seed produced from reciprocal crosses. The fact that no such differences were observed suggests that the genotype of the developing embryo is most likely the major point of control in the differential seed weight associated with *Sw4.1* alleles.

### Additive Interaction of the *L* and *S* Alleles

By comparing the seed weight values of the self-pollinated *L/L* and *S/S* NILs, it was estimated that the additive gene effect of *Sw4.1* is approximately 0.36 mg [(*LL*-*SS*)/2]. As a result, it is estimated that the genetic effect of a single “large-seed” (*L*) allele is to increase seed weight by 14% – a value very similar to what was reported previously [33]. Using these same parental values, as well as data from the F1 seed lots, it was also possible to calculate the degree of dominance (d/a or *k*) for interaction of the *L* and *S* alleles. It is thus estimated that the alleles interact in a largely additive manner (d/a = −0.21). This result suggests that the change in seed size affected by the two alleles is not due to a loss-of-function (e.g. deletion) at the locus, in which case a dominant-recessive gene action would be observed. This result is consistent with results from the gene expression experiments to be presented later.

### Timing of *Sw4.1* Effects during Seed Development

**Figure 2:**
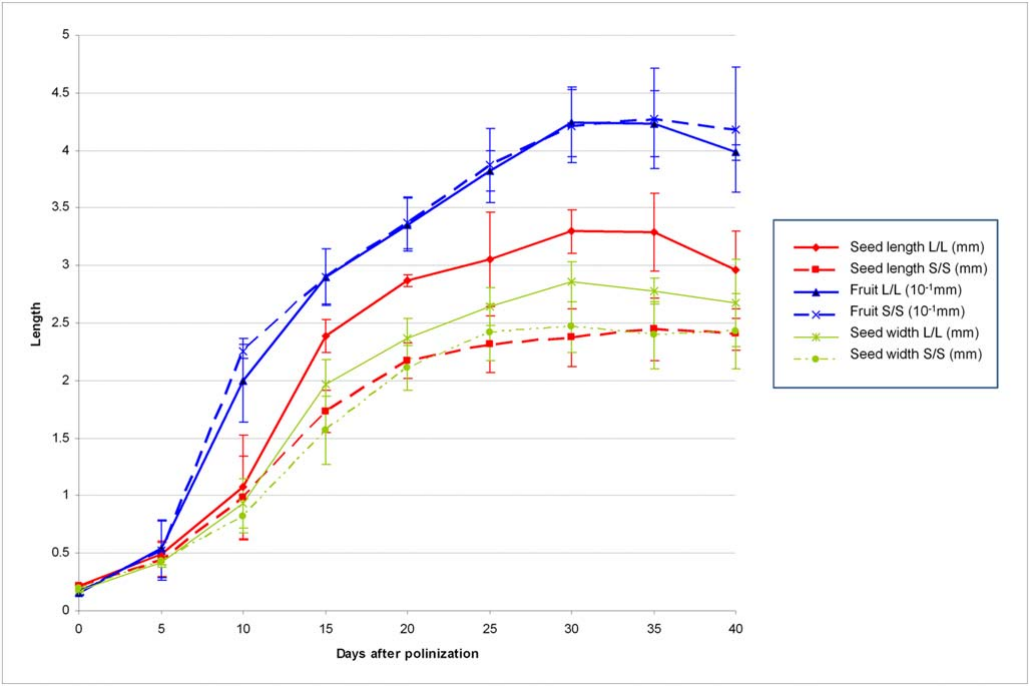
Plots for developmental changes in fruit length, seed width and seed length for the *L/L* and *S/S* NILs. Bars indicate the standard errors.

In an effort to identify the time during development at which *Sw4.1* alleles modulate seed size, a comparative developmental study was conducted on the large-seeded (*L/L*) NIL and small-seeded (*S/S*) NIL. Fruit size was also measured to determine whether *Sw4.1* may also affect this character. Developmental plots for the two NILs are shown in Figure 2. No differences were observed for the NILs with respect to fruit size at any time during development. It is therefore concluded that *Sw4.1* alleles specifically modulate changes in seed size, and that these changes are not the indirect effect of modulations in fruit size. These results are consistent with the earlier showing that the *Sw4.1* effect on seed size is exerted largely through the genotype of the zygote and not the maternal plant.

Beginning at 10 days after pollination (DAP), seed size (as measured by width and length) was consistently greater for the *L/L* NIL than for the *S/S* NIL. For example, seeds from the *L/L* NIL were on average 23% longer than those of the *S/S* NIL at 15 DAP (*P* = 0.008, Figure 2). At maturity, seeds of the *L/L* NIL were 17% longer than seeds of the S/S NIL (*P* = 0.001). Overall, *L/L* NIL showed the greatest change in seed size, relative to the *S/S* NIL, in the period from 10 DAP to 15 DAP, suggesting that *Sw4.1* exerts its largest differentiating effect during this early period of seed development. In an effort to determine the stage of embryo development corresponding to this critical period, paraffin cross sections were prepared from seeds at 10, 15 and 20 DAP. Microscopic examinations of the sections revealed that the embryos are globular at 10 DAP and torpedo-shape to curving at 15 DAP. These stages are similar to those previously reported by Lersten [35] and correspond to the initiation of rapid deposition of starch and lipids into the endospermic cells.

### *Sw4.1* Exerts Equal Effects on the Size of Both the Embryo and Endosperm but Does Not Affect Seed Viability or Germination Rate

An examination of sections of mature seed from the *L/L* and *S/S* NILs revealed that the *L/L* NIL produces seed that are increased with respect to the size of both the embryo (*P* = 0.014) and endosperm (*P* = 0.002) relative to those of the S/S NIL. However, no difference was observed between the two NILs with respect to the ratio of endosperm to embryo (*P* = 0.956). In both NILs the embryo accounts for the largest portion of the total seed with an embryo∶endosperm ratio of 4∶1. These results suggest that *Sw4.1* modulates changes in the amount of both embryo and endosperm tissue during seed development.

Germination tests of the *L/L* and *S/S* NILs revealed that both give rise to highly viable seed (germination rates of 97%±3.1 versus 96.3%±2.1, respectively). Therefore, while *Sw4.1* affects seed size, it appears to have no detectable affect on seed viability as measured by germination percentage. Another way to characterize seed viability is by germination rate. Germination rate is defined as the speed of germination, which is often associated with seedling vigor [36]. When the *L/L* and *S/S* NILs were subjected to germination rate tests, no difference was observed (*P* = 0.106). However, since these tests were done under laboratory conditions, we cannot rule out the possibility that allelic variation at *Sw4.1* does affect seed germination under natural or field conditions.

### High-Resolution Mapping of *Sw4.1*

**Figure 3:**
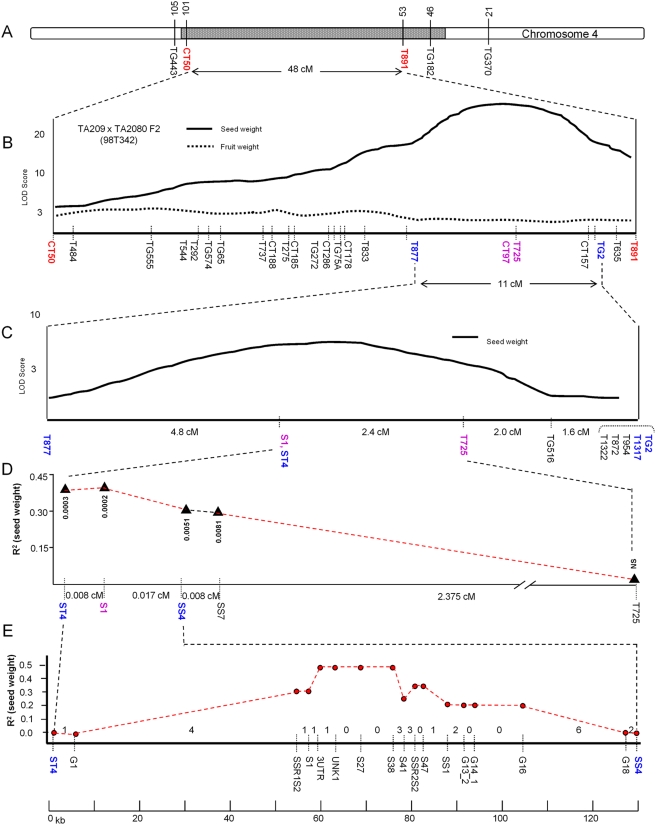
High resolution mapping of *Sw4.1* QTL on tomato chromosome 4. (A) TA2080 *S/S* NIL showing the introgression region from *S. pimpinellifolium* containing *Sw4.1* QTL (shaded). (B) *Sw4.1* mapping of F_2_-population of 150 individuals (TA209 – *L/L* NIL×TA2080 – *S/S* NIL) within a 48 cM region. (C) *Sw4.1* mapping within a 11 cM region from 140 individuals from a F_2_ heterozygous individual (98T342-95). (D) *Sw4.1* mapping within a 2.4 cM region from recombinants selected from 1,000 seeds from F_2_ heterozygous individuals. The S1 marker was then used to isolate and sequence the 130 kb BAC clone LE_HBa0077O05. (E) *Sw4.1* mapping within the ∼130 kb BAC LE_HBa0077O05 from recombinants selected from 9,000 seeds from F_2_ heterozygous individuals. The numbers between markers represent the number of crossover events in each interval.

A high-resolution genetic mapping study was conducted in an effort to establish the molecular basis for *Sw4.1*. This study was facilitated by the availability of the *L/L* and *S/S* NILs from which large F2 mapping populations could be derived. The first such F2 population was screened with the markers CT50 and T891 which flank the *Sw4.1* QTL (Figure 3A). DNA from individuals showing recombination between these two markers was further screened by an additional 23 markers within the region, allowing the *Sw4.1* QTL to be resolved to an 11 cM interval between markers T877 to TG2 (Figure 3B). Within this interval the highest LOD score was observed for marker CT97, 53% of the variation in seed weight was associated with this marker. To further resolve the position of *Sw4.1*, 140 additional individuals, derived from a F2-heterozygous individual, were then screened for all markers (including 9 additional markers) in the 11 cM interval. From this mapping, *Sw4.1* was further resolved to a 7 cM interval between marker T877 and T725 (Figure 3C). Within this interval, seed weight showed the strongest association with marker S1. The position of *Sw4.1* was further delimited to a 2.4 cM interval (between markers ST4 and T725) by the screening of an additional 1,000 progeny (Figure 3D). The S1 marker again showed the strongest association with seed weight explaining 41% of the seed weight variation.

**Figure 4:**
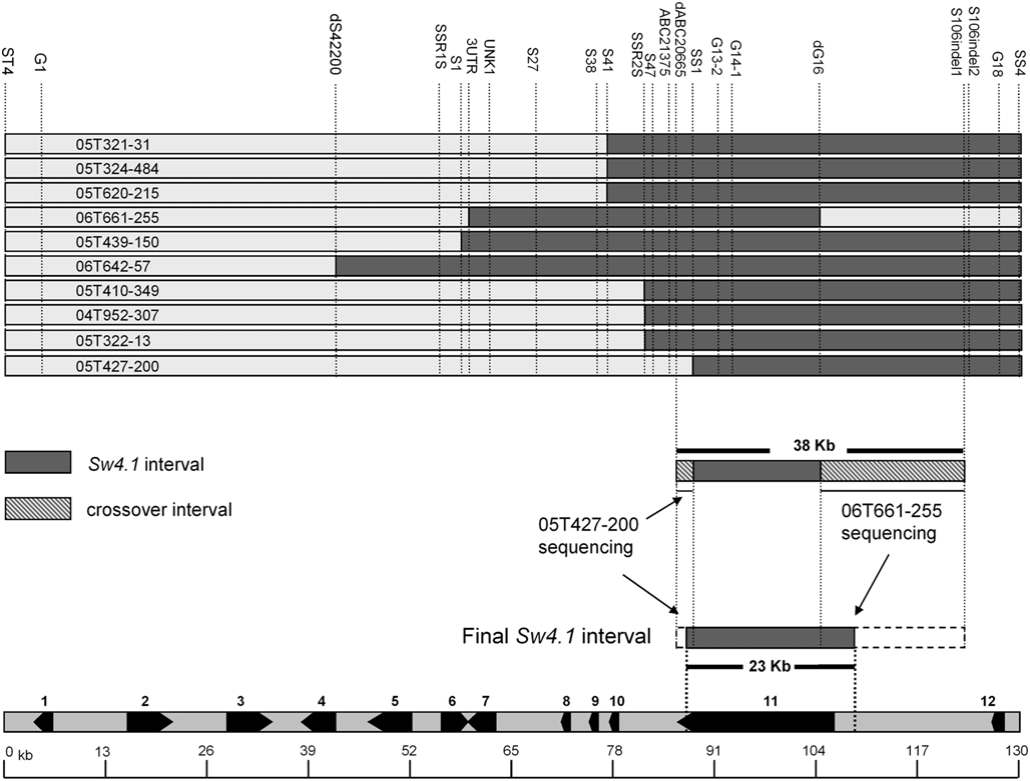
Results from progeny testing of key recombinants to delineate the position of *Sw4.1* in BAC. Shaded area indicates where *Sw4.1* QTL is assigned in each recombinant based on progeny tests (see Table 1). Cumulative results pinpointed *Sw4.1* to 38 kb interval. Sequencing of two key flanking recombinants (05T427-200 and 06T661-255) further delineated *Sw4.1* to a 23 kb interval containing single gene in BAC (gene 11, ABC transporter) (bottom).

The S1 marker was then used to isolate and sequence a 130 kb BAC (LE_HBa0077O05) from the *Sw4.1* region of chromosome 4. The markers SS4 and ST4, derived from the end sequence of this BAC, were subsequently used to screen an additional 9,000 F2 plants and identify 25 recombinants within the BAC interval. However, sufficient seed for analysis was obtained for only 13 of the recombinant individuals. The R^2^-plot for the seed weight association among the 13 recombinants indicated that the cause of the *Sw4.1* QTL resides within the central portion of this BAC (Figure 3E). To gain further precision on the location of *Sw4.1* within the BAC, selfed progenies from 10 selected recombinants were examined. These individuals contained crossovers between markers SSR1S2 and G18 (∼73 kb apart) - where the maximum marker association with seed weight was localized by fine mapping (Figure 3E, 4). A comparison of seed weight from homozygous recombinant and non-recombinant progeny allowed positioning of the *Sw4.1* QTL relative to the crossover point in each stock (Table 1, Figure 4).

**Table 1:** Progeny analysis of 10 heterozygous individuals with recombination within the BAC.

F3 recombinant	pedigree	Recombinant/ non-recombinant	A	B	C	D	E	F	G	H	I	J	K	L	M	N	O	P	Q	R	S	T	U	V	W	X	n	SWE (mg)	One tail t-test	P value
05T321-31	07T542	R	3	3	3	3	3	3	3	3	3	***1***	***1***	***1***	***1***	***1***	***1***	***1***	***1***	***1***	***1***	***1***	***1***	***1***	***1***	***1***	5	2.338	NR>R	0.105
		NR	1	1	1	1	1	1	1	1	1	***1***	***1***	***1***	***1***	***1***	***1***	***1***	***1***	***1***	***1***	***1***	***1***	***1***	***1***	***1***	6	2.552		
05T324-484	07T543	R	3	3	3	3	3	3	3	3	3	***1***	***1***	***1***	***1***	***1***	***1***	***1***	***1***	***1***	***1***	***1***	***1***	***1***	***1***	***1***	6	2.419	R>NR	0.039
		NR	3	3	3	3	3	3	3	3	3	***3***	***3***	***3***	***3***	***3***	***3***	***3***	***3***	***3***	***3***	***3***	***3***	***3***	***3***	***3***	6	1.992		
05T620-215	07T544	R	3	3	3	3	3	3	3	3	3	***1***	***1***	***1***	***1***	***1***	***1***	***1***	***1***	***1***	***1***	***1***	***1***	***1***	***1***	***1***	6	3.121	R>NR	0.011
		NR	3	3	3	3	3	3	3	3	3	***3***	***3***	***3***	***3***	***3***	***3***	***3***	***3***	***3***	***3***	***3***	***3***	***3***	***3***	***3***	5	2.5		
06T661-255	07T540	R	1	1	1	1	1	***3***	***3***	***3***	***3***	***3***	***3***	***3***	***3***	***3***	***3***	***3***	***3***	***3***	***1***	1	1	1	1	1	6	2.458	NR>R	0.004
		NR	1	1	1	1	1	***1***	***1***	***1***	***1***	***1***	***1***	***1***	***1***	***1***	***1***	***1***	***1***	***1***	***1***	1	1	1	1	1	5	3.075		
05T439-150	07T541	R	3	3	3	3	***1***	***1***	***1***	***1***	***1***	***1***	***1***	***1***	***1***	***1***	***1***	***1***	***1***	***1***	***1***	***1***	***1***	***1***	***1***	***1***	6	2.664	NR>R	0.21
		NR	1	1	1	1	***1***	***1***	***1***	***1***	***1***	***1***	***1***	***1***	***1***	***1***	***1***	***1***	***1***	***1***	***1***	***1***	***1***	***1***	***1***	***1***	6	2.795		
06T642-57	07T539	R	1	1	***1***	***3***	***3***	***3***	***3***	***3***	***3***	***3***	***3***	***3***	***3***	***3***	***3***	***3***	***3***	***3***	***3***	***3***	***3***	***3***	1	1	7	2.725	NR>R	0.01
		NR	1	1	***1***	***1***	***1***	***1***	***1***	***1***	***1***	***1***	***1***	***1***	***1***	***1***	***1***	***1***	***1***	***1***	***1***	***1***	***1***	***1***	1	1	6	3.072		
05T410-349	07T545	R	3	3	3	3	3	3	3	3	3	3	***1***	***1***	***1***	***1***	***1***	***1***	***1***	***1***	***1***	***1***	***1***	***1***	***1***	***1***	7	2.919	NR>R	0.42
		NR	1	1	1	1	1	1	1	1	1	1	***1***	***1***	***1***	***1***	***1***	***1***	***1***	***1***	***1***	***1***	***1***	***1***	***1***	***1***	7	2.956		
04T952-307	07T546	R	3	3	3	3	3	3	3	3	3	3	***1***	***1***	***1***	***1***	***1***	***1***	***1***	***1***	***1***	***1***	***1***	***1***	***1***	***1***	6	2.776	R>NR	0.001
		NR	3	3	3	3	3	3	3	3	3	3	***3***	***3***	***3***	***3***	***3***	***3***	***3***	***3***	***3***	***3***	***3***	***3***	***3***	***3***	5	1.891		
05T322-13	07T547	R	3	3	3	3	3	3	3	3	3	3	***1***	***1***	***1***	***1***	***1***	***1***	***1***	***1***	***1***	***1***	***1***	***1***	***1***	***1***	4	2.942	R>NR	0.003
		NR	3	3	3	3	3	3	3	3	3	3	***3***	***3***	***3***	***3***	***3***	***3***	***3***	***3***	***3***	***3***	***3***	***3***	***3***	***3***	3	2.497		
05T427-200	07T548	R	3	3	3	3	3	3	3	3	3	3	3	3	3	3	***1***	***1***	***1***	***1***	***1***	***1***	***1***	***1***	***1***	***1***	6	2.84	R>NR	0.001
		NR	3	3	3	3	3	3	3	3	3	3	3	3	3	3	***3***	***3***	***3***	***3***	***3***	***3***	***3***	***3***	***3***	***3***	4	2.095		
																X	X	X	X	X	X	X								

“1” indicates homozygous for corresponding marker from *L/L* NIL. “3” indicates homozygous for corresponding marker from *S/S* NIL. SWE = seed weight. In italic and bold is the region indicated by the one-tail t-test of either recombinant versus non-recombinant (R>NR) or non-recombinant versus recombinant (NR>R) for the positioning of the *Sw4.1* QTL. Shown at bottom (X) is the consensus region for the location of *Sw4.1* based on progeny tests from all 10 recombinants. Marker A = ST4; B = G1; C = dS4220; D = SSR1S2; E = S1; F = 3UTR; G = UNK1; H = S27; I = S38; J = S41; K = SSR2S2; L = S47; M = ABC21375; N = dABC20665; O = SS1; P = G13-2; Q = G14-1; R = G16; S = S106indel1; T = S106indel2; U = G18; V = SS4; W = SS7; X = T725.

Two independent crossover events (05T427-200 and 06T662-255) delineated the cause of the *Sw4.1* QTL to a 38 kb interval extending from markers dABC20665 to S106 indel1 (Table 1, Figure 4). The exact position of the crossover in each of these recombinant stocks was established by PCR sequencing through the crossover boundary region for each stock. As a result, the cause of the *Sw4.1* QTL could be further narrowed to a smaller, 23 kb interval (Figure 4). Based on annotation, this 23 kb interval contains a single gene encoding a putative ATP binding cassette (ABC) transporter protein (gene 11 in Figure 4).

### Identifying the Arabidopsis Ortholog to the *Sw4.1* ABC Transporter Gene

ABC transporters represent a super family of ATP-binding cassette proteins found in a wide range of species [37]. They are used in transmembrane transport of diverse substances – including peptides, sugars, lipids, heavy metal chelates, polysaccharides, alkaloids, steroids, inorganic acids and glutathione conjugates [38]–[42]. The Arabidopsis genome contains at least 129 ABC transporter-like genes, and a number have already been investigated with regards to function [43]. As an aid to annotation of the tomato ABC transporter gene, and in the hope of gaining possible insights into its function, an effort was made to determine which of these Arabidopsis ABC transporter gene(s) might be orthologous to the tomato gene associated with *Sw4.1*.

**Figure 5:**
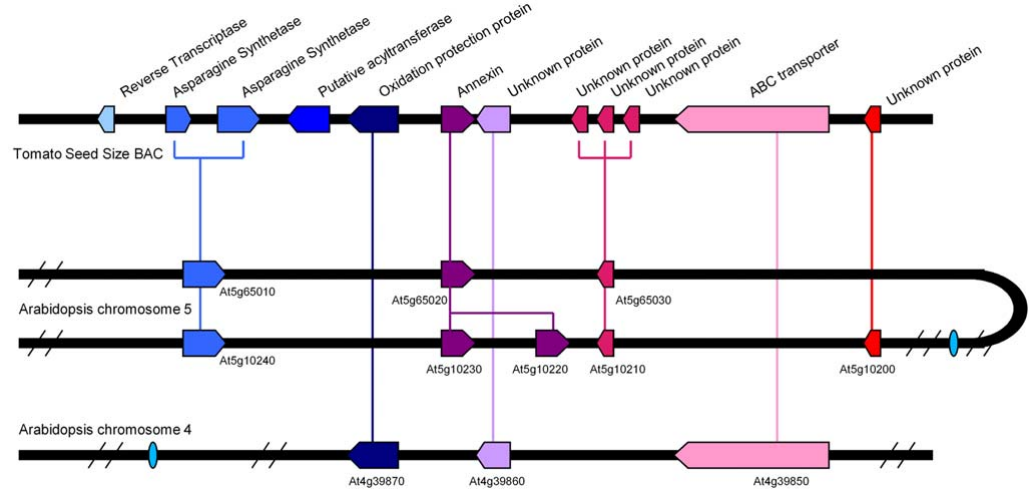
Relationship of genes in tomato BAC containing *Sw4.1* and corresponding syntenic regions in Arabidopsis genome.

Synteny has proven a very powerful method for establishing orthology [44]. A Reciprocal Best Match (RBC) approach [45] was thus used to identify microsyntenic genomic region(s) between Arabidopsis and tomato for the *Sw4.1* region. With this approach, an effort was made to identify the putative orthologs of the 12 genes annotated in the tomato BAC containing the ABC transporter gene (Table S1). As a result, three microsyntenic blocks were identified in Arabidopsis – two blocks located on chromosome 5 and one on chromosome 4 (Figure 5). Only the syntenic block on chromosome 4 contains an ABC transporter (At4g39850) (Figure 5). It is therefore concluded that the Arabidopsis ABC transporter gene At4g39850 is orthologous to the tomato ABC transporter associated with *Sw4.1*. It is worth noting that At4g39850 is located at the same chromosomal position as a QTL associated with variation in both seed length and width in a cross between Arabidopsis ecotypes, raising the possibility that this gene may also underlie natural variation in seed size in this species [16],[46].

**Figure 6:**
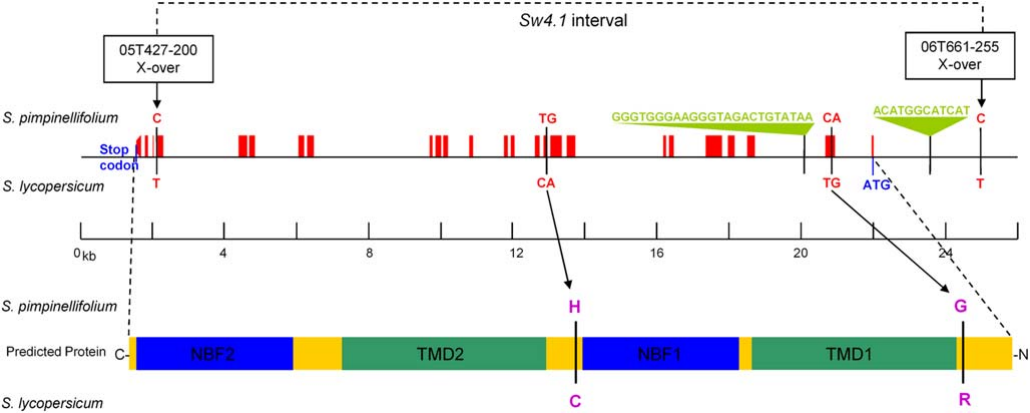
Annotation of tomato ABC transporter gene associated with *Sw4.1* QTL. Exons are shown in red. Conserved functional domains are shown in blue, ATP-binding cassette (ABC) or nucleotide binding folds (NBFs), and green, hydrophobic integral membrane domains (TMDs).

Sequence alignment of Arabidopsis At4g39850 cDNA with the genomic sequences of both the *L* allele (*S. lycopersicum*) and *S* allele (*S. pimpinellifolium*) allowed the prediction of intron and exon boundaries in the tomato gene (Figure 6). It also allowed prediction of the full-length tomato ABC transporter protein, which is characterized by 4 functional domains, two of which are ATP-binding cassette (ABC) or nucleotide binding folds (NBFs) and two of which are hydrophobic integral membrane domains (TMDs) (Figure 6) [43]. While both the length of coding region and the intron positions are highly conserved between the tomato and Arabidopsis orthologs, the introns are more variable in length and are generally longer in tomato than Arabidopsis (data not shown).

Several T-DNA insertion mutants have been isolated for the Arabidopsis ortholog At4g39850. Some of these mutants affected seed size [47]. However, the Arabidopsis mutants were associated with an increase in seed size, whereas in tomato the RNAi transgenics produced smaller seeds. Another difference between Arabidopsis and tomato, is that some of the Arabidopsis mutants also affected seed germination, whereas no germination effects were observed for *Sw4.1* in tomato. Further, one of the mutants (*cts-2*) produced seeds with significant higher levels of fatty acids. These results led to the suggestion that the Arabidopsis ABC transporter protein might be involved, not only in lipid metabolism during germination, but also in lipid accumulation during seed development – possibly explaining why the mutants produced larger seeds [47]. In this regard, it is worth noting that in tomato the *Sw4.1* QTL produces its largest effects during the stages of seed development associated with lipid deposition (see previous section, Figure 2). Thus, it is possible that the tomato ABC transporter gene modulates seed size by controlling the accumulation of lipids during seed development.

### Testing the Effects of the ABC Transporter Gene on Seed Size via RNAi Transformation Experiments

Transformation experiments were used to test whether the ABC transporter gene has the ability to modulate seed size. This gene is quite long (due to many introns) – spanning more than 20 kb from the start to stop codon (Figure 6).

The large size of the gene, absence of efficient enzymatic sites for cloning and lack of a full-length cDNA, precluded complementation analysis with the full-length genomic copy of the gene. Therefore, a gene silencing approach, via RNAi hairpin formation, was employed as an alternative strategy for testing the potential role of the ABC transporter gene in determining seed size.

Efficient post-transcriptional silencing has been reported when 3′UTR regions are targeted for RNAi machinery [48],[49]. Hence, a 278 bp fragment from the 3′UTR of the ABC transporter gene was inserted in the pHELLSGATE2 (Invitrogen) binary vector, the pSP13-1 construct (Figure S1). Sequence specificity was assessed by blasting the 278 bp fragment against the tomato unigene database in the SGN website (www.sgn.cornell.edu). The retrieval of a unique unigene that corresponds to this ABC transporter gene suggests this sequence is specific to this gene and therefore there would present a low risk to silence other genes in the same ABC transporter family. This construct was transformed into both the *L/L* and *S/S* NILs. Multiple independent T_0_ and non-transgenic controls were then analyzed for both NIL sets. *L/L* transformants were highly fertile, yet produced seeds weighing on average 38% less than those from the non-transgenic controls (*P* = 0.03, Table 2). *S/S* transformants also produced smaller seed (11% less heavy) than the non-transgenic controls, however the statistical difference did not quite reach statistical significance (*P* = 0.09, Table 2). It is worth noting that transformation/complementation with two other genes from the BAC (annexin and a gene of unknown function – genes number 6 and 7, respectively, in Figure 4) did not show statistical difference between transgenic and non-transgenic plants (data not shown). The transformation experiments thus appear to corroborate the results from high-resolution mapping – both pointing to the ABC transporter gene as the cause of the *Sw4.1* QTL.

**Table 2:** Summary of transgenic silencing of ABC transporter gene in *L/L* and *S/S* NILs based on RNA interference.

NIL Plant	Transgenic Status	N	Seed Weight (mean±SE – mg)	*P* value
**L/L**	Transgenic	10	2.016±0.345	0.031
	Non-transgenic	3	3.235±0.522	
**S/S**	Transgenic	10	2.262±0.495	0.093
	Non-transgenic	2	2.529±0.132	

### Allelic Polymorphisms in the ABC Transporter Gene

Comparing the sequence of the *L* and *S* allele, within the 23 kb *Sw4.1* interval, revealed 79 SNPs and single indels located in introns, 10 in the promoter region and 6 in exons (Figure S2). In the exons, 2 non-synonymous changes were observed (Figure 6). Either of these might be causal to the phenotypic effects rendered by the *L* and *S* alleles. However, neither substitution is located in a conserved functional domain (e.g. nucleotide binding folds or hydrophobic integral membrane domains) (Figure 6). Among the polymorphisms in non-coding regions, a few are worth mentioning as possible causal candidates for the *Sw4.1* QTL. One is a 12 nt indel approximately 1.5 kb upstream in the 5′ promoter and the second, a 24 nt indel in the first intron (Figure 6). Either of these indels might cause a change in expression – as could the many other small nucleotide differences observed in non-coding regions of the two alleles.

### Expression of ABC Transporter Gene during Seed Development

**Figure 7:**
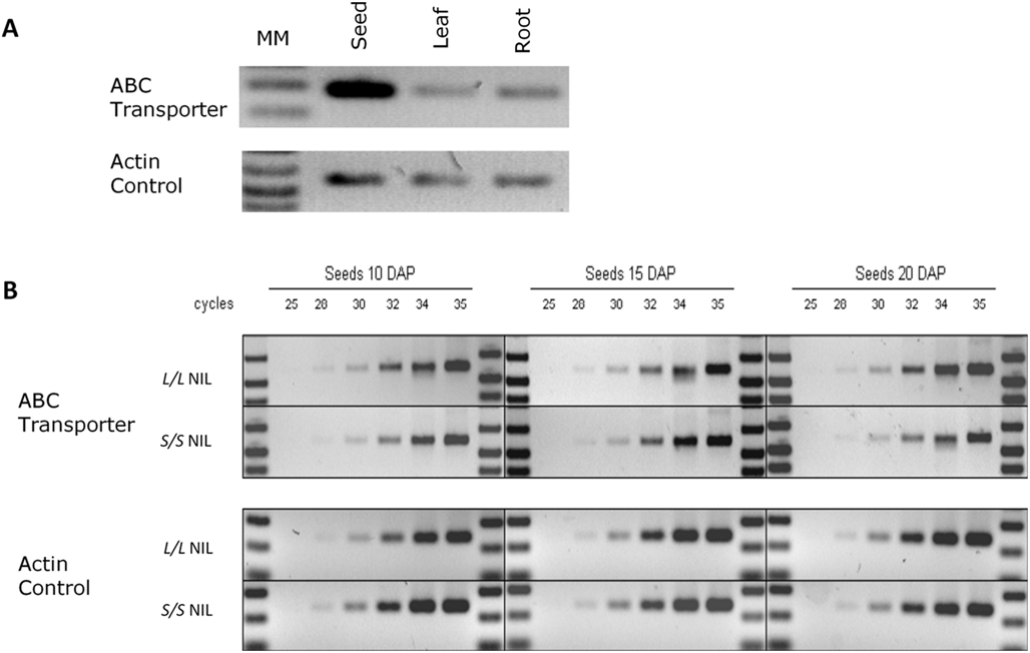
RNA expression studies of tomato ABC transporter gene. (A) Comparison of expression in seeds, leaves and roots using semi-quantitative RT-PCR. Actin gene used as a comparative control. (B) Semi-quantitative RT-PCR of ABC transporter gene during seed development comparing *L/L* and *S/S* NILs.

In an effort to determine the expression pattern of the ABC transporter gene, and especially whether the *L* and *S* allele differ in regulation/expression during seed development, a set of semi-quantitative RT-PCR experiments were conducted. The first experiment revealed that this gene is expressed at high levels in seeds, and lower levels in shoots and roots (Figure 7A). High expression of the ABC transporter gene in developing seeds is further evidence that this gene is the cause of the *Sw4.1* QTL. The subsequent experiment compared expression of the ABC transporter gene in both the *L* and *S* NILs at 10, 15 and 20 DAP – the time when the major change in seed size is observed (see previous section) (Figure 7B). Two conclusions can be drawn from this second experiment. First, the ABC gene is expressed in both the *L* and *S* NILs – ruling out a loss-of-function as the cause of seed size variation associated with *Sw4.1.* Second, the failure to detect any major change in expression of the ABC transporter gene between the *L* and *S* NILs during seed development would seem to rule out a gross change in the regulation as the cause of the *Sw4.1* QTL (Figure 7B). However, we cannot exclude the possibility of a difference in translational/post-translational regulation or small changes in spatial or temporal regulation as the cause of QTL effect. For example, in tomato it was previously shown that a modest change in the timing of allele expression can cause major QTL effects on fruit size [50].

### Conclusions

The results presented herein point to natural variation in an ABC transporter gene as a major cause of the change in seed size that differentiates the cultivated tomato from related wild species. While prior studies in a number of plants have reported the effects of induced mutations on seed size, this example is among the few in which the cause of changes in seed size in nature populations has been pinpointed. Further, the ortholog to this gene in Arabidopsis has been identified through homology/synteny. Results from QTL mapping and mutagenesis studies are consistent with this gene also playing a role in determining seed size in Arabidopsis. However, further studies are required to clearly establish whether this ABC transporter gene operates in a similar manner in both tomato and Arabidopsis. Also, it remains to be established whether variation in ABC transporter genes is a major cause of seed size variation in natural populations of Arabidopsis or other plant species. Having identified the cause of the *Sw4.1* QTL in tomato may open he door to addressing these questions in the future.

## Materials and Methods

### Plant Material and Genetic Markers

Two *Solanum lycopersicum* nearly isogenic lines (NILs), with alternative alleles at the *Sw4.1* locus, were the origin of all genetic stocks used in this study. TA209 carries the large-seeded (*S. lycopersicum*) allele at *Sw4.1* locus. TA2080 is isogenic with TA209, but carries a 55–84 cM segment of chromosome 4 of *S. pimpinellifolium* LA1589 (Figure 3A) containing the small-seeded allele of *Sw4.1* [13],[33]. TA2080 was developed via marker assisted selection during 5 sequential backcrosses of LA1589 into TA209 followed by a single selfing generation (BC_5_S_1_). An F2 population, segregating for *Sw4.1*, was then developed from a cross between TA209 and TA2080. A derived, shorter introgression NIL, derived from TA2080, was used for the reciprocal cross experiments. Likewise, the *S/S* NIL (TA3820) used for transformation experiments was also a shorter derivative of TA2080. Detailed description of the construction of both derived subNILs can be found in Orsi [46]. The sequence of all markers used in this study can be found in Table S2.

### Reciprocal Cross and Gene Action Experiments

Plants were grown in pots in the greenhouse in 12 randomized blocks. Each block was comprised of the following *Sw4.1* genotypes and type of pollination: *L/L*: selfing, *S/S*: selfing, *L/S*: selfing; *L/L*: used as the female in crosses to *S/S*, *S/S*: used the female in crosses to *L/L*. Each plant was either selfed or crossed manually. At maturity, 5 normal fruit (no blossom end rot, non-parthenocarpic) were harvested from each plant and the seed extracted. The fruit were weighed and the average weight per fruit was recorded. From a pool of seeds from 5 fruits, 50 healthy seeds were randomly sampled. From these, an average seed weight was calculated for each plant. A comparison of the least squares means was performed using the adjustment for multiple comparisons Tukey-Kramer (SAS enterprise guide 3.0).

### Developmental Analyses

Effects of *Sw4.1* on seed development and fruit size: Ten pairs of *L/L* and *S/S* NIL plants were grown in the greenhouse. Two to three fruits were harvest from each plant at each of the following stages: anthesis (0 days after pollination –DAP), 5 DAP, 10 DAP, 15 DAP, 20 DAP, 25 DAP, 30 DAP, 35 DAP and 40 DAP. Because of the microscopic size of ovule and developing seeds, it was not possible to collect mass (weight) data. Instead, all traits were recorded as spatial metrics (e.g., length, width). Both fruit and seed were scanned using a HP ScanJet (1200 dpi). Length and width measurements were then extracted from the images using the software Tomato Analyzer Version v.1.2 [51]. A second independent experiment, of identical design, was then conducted. However, based on results from the first experiment, fruit were collected only at 10 DAP, 15 DAP and 20 DAP – periods associated with most rapid changes in seed development. For the verification of seed developmental stages, seeds from 10, 15 and 20 DAP were fixed, dehydrated and embedded in paraplast (Sigma) as described by [52].

Role of *Sw4.1* in determining the proportion of embryo to endosperm in mature seed: Five pairs of *L/L* and *S/S* individuals were grown in the greenhouse. Five mature fruits were harvested from each individual and the seeds extracted. A random sample of 10 normal seeds was then drawn from each individual. Each individual seed was then dissected longitudinally into halves and the images digitalized under a dissecting scope (ZeissStemi 2000-CS attached to 3CCD camera MTI) using the software Scion Image (www.scioncorp.com). The areas of the entire seed, embryo and endosperm were manually delineated and measured using the software ImageJ 1.31 v (http://rsb.info.nih.gov/ij/).

### Seed Germination and Viability Experiments

Ten pairs of *L/L* and *S/S* individuals were grown in the greenhouse. One hundred normal seeds from a pool of 10 fruits of each genotype were germinated in Petri dishes on filter paper saturated with distilled water. The seeds were scanned prior to the germination process for seed length measurement (Tomato Analyzer v.1.2). The number of germinated seed was recorded on a daily basis until no additional seed germination was obtained. For these experiments, a seed was considered germinated once the root tip had emerged. Tests of heterogeneity on the number of germinated seeds at days 3, 4 and 5 (when the majority of seed germination was observed for all the genotypes) were performed for the detection of possible differences in germination rate between NILs (Minitab 15).

### Progeny Testing of Selected Recombinants

The genetic stocks used for the progeny analysis were derived from sub-NILs selected from each selected recombinant within the BAC (Figure 4). Ten homozygous recombinant and ten homozygous non-recombinant individuals were selected, via marker analysis, from selfed seed of selected recombinants (Table 1, Figure 4). The selected progeny were grown in a completely randomized design in the greenhouse. From each plant, seeds were extracted from 5 fruits and pooled. From each pool, 50 normal seeds were randomly selected and weighed. For each family, statistical comparisons for seed weight were made between the 10 recombinant and 10 non-recombinant progeny from each family using a one-tailed t-test (Minitab 15) (Table 1).

### BAC Annotation and Comparison with the Arabidopsis Genome

BAC LE_HBa0077O05, isolated with marker probe S1, was annotated using the automated annotation tools developed by SGN and refined manually through BLAST searches against EST libraries (Solanaceae, coffee, Arabidopsis). Arabidopsis genes, putatively orthologous to genes in the tomato BAC, were identified using the Best Reciprocal Matches (RBM) in BLAST comparisons [45]. Based on these putative orthologs, it was possible to identify region in the Arabidopsis genome showing conserved microsynteny with the *Sw4.1* region of tomato chromosome 4.

### Sequencing of *Sw4.1* Interval from *S. pimpinellifolium* and Annotation of Polymorphisms between *S. lycopersicum* and *S. pimpinellifolium*

A 38 kb segment, delimited by markers dABC20665 and S106indel1 and known from progeny analyses to encompass the *Sw4.1* QTL, was also sequenced via PCR from the genome of *S. pimpinellifolium* LA1589 – the small-seeded parent of the original mapping population. The objective of the sequencing was to identify polymorphisms that might be causal to the *Sw4.1* QTL. Sequence analysis was performed with DNASTAR Lasergene software and alignments with BioEdit version 7.0.9.0 using ClustalW.

### Sequencing Tecombinants in the 38 kb *Sw4.1* Interval to Pinpoint the Exact Crossover Points

In order to further narrow the *Sw4.1* interval, PCR-based sequencing was performed on the two recombinant individuals that define the left (05T427-200) and right (06T661-255) side of the 38 kb interval. By sequencing across the exact crossover point in each recombinant, it was possible to narrow the location of the *Sw4.1* QTL to a 23 kb interval (Figure 4).

### Transformation Experiments

#### RNAi transgene construction

A 278 bp fragment from the 3′UTR of the ABC transporter gene orthologous to At4g39850 was inserted in the binary vector pHellsGate 2 (Invitrogen) [53] for the generation of a hairpin construct for the RNAi mechanism induction and gene silencing in *L/L* and *S/S* NILs. The schematic representation of plasmid construction is shown in Figure S1. The pHELLSGATE vector was designed such that a single PCR product from primers with the appropriate attB1 and attB2 sites would be recombined into it simultaneously to form the two arms of the hairpin [53]. The recovery of successful recombination (insertion) of both arms of the hairpin was ensured by ccdB genes, which were replaced by the arm sequences (ABC transporter fragment). CcdB gene is lethal in standard *E. coli* strains such as DH5a, strain that was used for plasmid cloning. The intron retention was ensured by the chloramphenicol-resistance gene (CAM) within the intron. The successful insertion for both arms of the hairpin was confirmed by sequencing analysis (data not shown). Recombinant pHELLSGATE constructs, called pSP13-1, were sent for transformation (Plant Science Initiative, University of Nebraska) for the direct transformation into Agrobacterium for transformation into the *L/L* and *S/S* NILs.

#### Experimental design and statistics

Transgenic analysis of the T_0_ generation: 10 transgenic *L/L* NILs, 3 non-transgenic *L/L* NILs, 10 transgenic *S/S* NILs and 2 non-transgenic *S/S* NILs were grown in greenhouse in a random design. Classification of plants as transgenic or non-transgenic was based on the amplification of the insert-vector using the primers AttB1-ABC and XhoI insert-vector for the sense insertion (403 bp) and Att1B1-ABC and XbaI insert-vector for the anti-sense insertion (400 bp). RNA levels for the ABC transporter gene were not measured in seeds from T_0_ plants since this would have precluded seed size measurements from the same plants. For efficient RNAi mechanism induction, both arms of the hairpin must be present as well as the intronic sequence. Therefore, amplification of both XbaI and XhoI were required for the assignment of transgenic plants. As a positive control for PCR amplification, S85 primers were included in each reaction. S85 amplifies 800 bp from the ABC transporter promoter region, which would be present in transgenic and non-transgenic plants. Five fruits of each plant were harvested and the seeds were extracted. 50–100 seeds were randomly selected from each pool and weighed. One tail t-tests, comparing transgenic and non-transgenic plants were then performed using Minitab 15.

### Analysis of ABC Transport Gene Transcript

#### Experimental design

Ten *L/L* NIL and ten *S/S* NIL individuals were paired and grown in greenhouse. Each flower was manually pollinated and the date of pollination noted. Seeds from fruits 10, 15 and 20 DAP were removed and immediately frozen in liquid nitrogen. Seeds at these stages were pooled from 5 healthy plants for the RNA extraction. Frozen seeds were ground to a fine powder in liquid nitrogen and total RNA was isolated using Trizol reagent (Invitrogen). The concentration of total RNA from each sample was determined from 100× diluted solution using spectrophotometry. One microgram of total RNA from each sample was treated with RNase-free DNaseI (amplification grade, Invitrogen). First-strand cDNA was synthesized by reverse transcription with oligo(dT)_16_ primer following manufacturer's protocol (Invitrogen). Another set of plants was grown and the procedure repeated as an independent experiment for replicate.

#### Semi-quantitative RT-PCR

ABC transporter transcript levels were detected by using semi-quantitative RT-PCR. The primers used for the amplification are S53R (5′ GGGAAGACGAACCAAATGAA 3′) and ABCp35R (5′ CGGGAACTAGGCGCTATACA 3′). These primers were designed based on the 3′end and 3′UTR of the gene. BLAST search against *A. thaliana* non-redundant database in NCBI and tomato EST database in SGN (www.sgn.cornell.edu) confirmed the uniqueness of this sequence and specificity to this ABC transporter gene. The internal control was actin TOM52 [54]. For the semi-quantitative approach, from a total reaction of 100 ul, 10 ul were collected at the end of each one of these cycles: 25, 28, 30, 32, 34 and 35.

## Supporting Information

Figure S1 Diagram of pSP13-1 construct used for RNAi based gene silencing of ABC transporter gene in transgenic experiments.
(0.75 MB TIF)

Figure S2 Detailed alignment of *L* and *S* alleles showing all polymorphisms in the 23 kb region encompassing the *Sw4.1* QTL.
(7.55 MB TIF)

Table S1 Annotation of the 12 genes contained in the tomato BAC LE_HBa0077O05.
(0.04 MB DOC)

Table S2 Markers for *S. lycopersicum* and *S. pimpinellifolium*: marker type, primer sequence, restriction enzyme and fragment size.
(0.15 MB DOC)
