# Supplementary figures and images for: Natural Variation in an ABC Transporter Gene Associated with Seed Size Evolution in Tomato Species
Source: PLoS Genet. 2009 Jan 23;5(1):e1000347. doi: 10.1371/journal.pgen.1000347 (PMC2617763; doi:10.1371/journal.pgen.1000347)

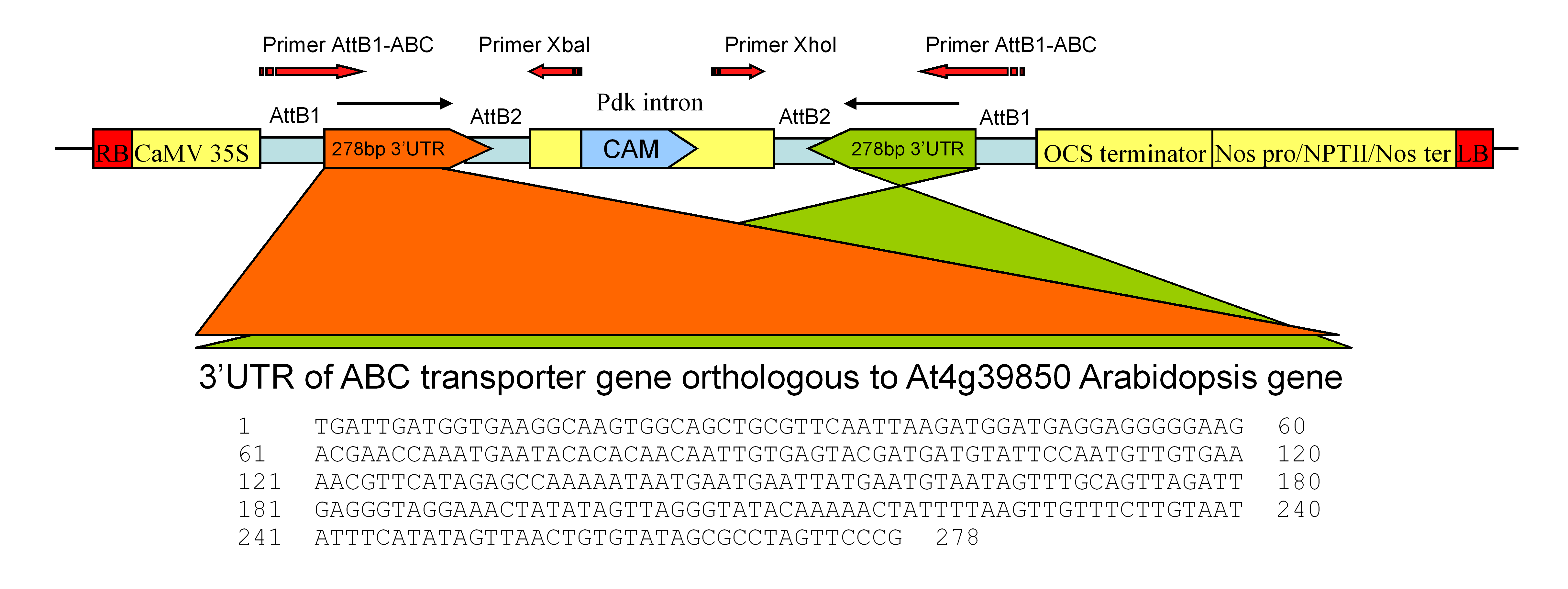

Supplement: Figure S1 — Diagram of pSP13-1 construct used for RNAi based gene silencing of ABC transporter gene in transgenic experiments. (0.75 MB TIF) [file pgen.1000347.s001.tif]
