# Supplementary material for: Natural Variation in an ABC Transporter Gene Associated with Seed Size Evolution in Tomato Species
Source: PLoS Genet. 2009 Jan 23;5(1):e1000347. doi: 10.1371/journal.pgen.1000347 (PMC2617763; doi:10.1371/journal.pgen.1000347)
Supplement: Table S1 — Annotation of the 12 genes contained in the tomato BAC LE_HBa0077O05. (0.04 MB DOC) [file pgen.1000347.s003.doc]

| **Order** | **Gene annotation** | **Strand** | **Start** | **End** | **Total length (bp)** |
| --- | --- | --- | --- | --- | --- |
| 1 | Reverse Transcriptase | reverse | 5465 | 3410 | 2055 |
| 2 | Asparagine Synthetase | forward | 15376 | 21460 | 6084 |
| 3 | Asparagine Synthetase | forward | 28060 | 34413 | 6353 |
| 4 | Putative Acyltransferase | reverse | 42526 | 37914 | 4612 |
| 5 | Oxidation Protection Protein | reverse | 51905 | 48714 | 3191 |
| 6 | Annexin | forward | 56003 | 59278 | 3275 |
| 7 | Unknown Protein | reverse | 62805 | 59854 | 2951 |
| 8 | Putative Protein | reverse | 72557 | 72010 | 547 |
| 9 | Putative Protein | reverse | 76536 | 75633 | 903 |
| 10 | Putative Protein | reverse | 78848 | 78337 | 511 |
| 11 | ABC transporter | reverse | 106990 | 86603 | 20387 |
| 12 | Unknown Protein | reverse | 128614 | 127463 | 1151 |
