# Supplementary material for: Natural Variation in an ABC Transporter Gene Associated with Seed Size Evolution in Tomato Species
Source: PLoS Genet. 2009 Jan 23;5(1):e1000347. doi: 10.1371/journal.pgen.1000347 (PMC2617763; doi:10.1371/journal.pgen.1000347)
Supplement: Table S2 — Markers for S. lycopersicum and S. pimpinellifolium: marker type, primer sequence, restriction enzyme and fragment size. (0.15 MB DOC) [file pgen.1000347.s004.doc]

| **Marker Name** | **Marker type** | **Name of forward primer** | **Primer sequence - Forward** | **Name of reverse primer** | **Primer sequence - Reverse** | **PCR (TM)** | **Restriction enzyme** | **Fragment size** |
| --- | --- | --- | --- | --- | --- | --- | --- | --- |
| 3UTR | dCAPS | 3utrF | TGACATGGAGCGTATCAAAGA | d3utrR | GAAGGCGAAAACAAAACACAACTCAACTCT | 60oC | TaqI | 270/300 |
| ABC20985 | CAPS | S53F | AACGTGGGTTTCTTACATGGAT | S53R | GGGAAGACGAACCAAATGAA | 60oC | MboII | 222/597 |
| ABC21375 | dCAPS | ABC21375F | GGTTGGACCTGGATCAGAGA | ABC21375R | CCCAATCCATAATGGTCCAA | 60oC | DpnII | 600/400 |
| ABC9070 | CAPS | S67F | AAACAACCACTGTGAGAGGAAA | S67R | TCTGGATCGCTATCCTCCTG | 60oC | BsrI | 750/780 |
| ABCpro | Indel/caps | ABCproF | ACTCAATTTTGATACCAATGTCACC | ABCproR1 | ACCCATCATACATGGCATCAT | 60oC | NlaIII | _/217 |
|  | Indel/caps |  |  | ABCproR2 | CGCAGTGCTGAAGTAGTGGA | 60oC | NlaIII | 470/482 |
| CT185 | CAPS | CT185F | GGCTCGGACAGAGGTTGATA | CT185R | ATAAAGGGTCGCCATTTTCC | 60oC | HindIII | 347/284 |
| CT50 | CAPS | CT50F | TTTGGGATTCAAGCTGGTTC | CT50R | GTAACACAGGCTGCTCCACA | 60oC | DdeI | 704/437 |
| d488 | dCAPS | d488F | TTAAATTTTTTGGATGTAAATCAAATATCG | 488R | TTTTGAAGCAGTTTTAGTCCAAT | 60oC | TaqI | 400/250 |
| dABC1200 | dCAPS | dABC1200F | TGGTATAATAGAGCCAAATAAGCAGGC | ABC1200R | CCAGCAGTAGTTTCCGCATA | 60oC | HaeIII | 350/400 |
| dABC20665 | dCAPS | dABC20665F | TGAATAAAAATGGCAGGTAGAAAAGGAAGC | ABC20665R | AACCCAACATCCAACCCTTT | 60oC | AluI | 350/400 |
| dABC6 | dCAPS | ABC6F1 | GGTGTAATGATGTCCACTTCAGAGA | dABC6R1 | ATCTAGCACGCAAAGGAACTATGACTCGA | 60oC | EcoRI | 530/500 |
| dABC7 | dCAPS | dABC7F2 | GGGTACCTAAAACAAGCACTAGAGCGAT | ABC7R2 | GTGGAAGTTTCCTTGGAACACTCT | 60oC | EcoRV | 500/530 |
| dG16 | dCAPS | G16F | TACCGCCCAGCTCACTTAAC | dG16R | TACTTTCTTATTGTCCTGCTATTGCTTGGC | 60oC | HaeIII | 400/370 |
| dS37500 | dCAPS | S37500F | TGCCACCAAGAGTAGGTAGTCC | dS37500R | TTGTGCATAAAGTGCATTCTGTATGGAAG | 60oC | HaeIII | 400/370 |
| dS42200 | dCAPS | S42200F | GGCCCTATGGAAGGAGAAGA | dS42200R | CTGTTGAACACATTATCAATCATCATGAT | 60oC | DpnI | 330/305 |
| dS6000 | dCAPS | dS6000F | AGTTTGTAATGCCAAGATACCTTCCTTCTCG | S6000R | TTTGGGTTGTGATGTGGAAA | 60oC | TaqI | 330/305 |
| dUnk1-5 | dCAPS | Unk5F | GATTAAATTCCAAGCAAAATGTGAC | dUNK5R | GTGACTTGTGTTGTTTGCTTTTT | 60oC | TaqI | 470/500 |
| dUnk2 | dCAPS | Unk2F2 | AATAGCAAGAAACATAGAACAAATAAGTCC | dUnk2R2 | CACTCACAATAACCCCTGCAATTACATGGC | 60oC | HaeIII | 470/500 |
| G1 | dCAPS | G1F2 | GGCACAAGATTTTTGCCAAT | dG1R | CTGGAACATTAGAGGAGGGAATTCCGGTCG | 60oC | TaqI | 400/370 |
| G13-2 | CAPS | G13_2F | TGCAAAGCAAGAGGTTGTTG | G13_2R | CATAGGCCTTGAAGCGAGTC | 60oC | HinfI | 750/500 |
| G14_1 | CAPS | G14_1F | TTTCCCGATGATGTGGCTA | G14_1R | CAAAAGTGGGGCTGAGATGT | 60oC | AflII | 750/500 |
| G18 | CAPS | G18F | CCTGTGCTCTTCTCCAAAGG | G18R | AACTTTTCGGCGTGATCTCT | 60oC | EcoRI | 250/100 |
| pSP13-1 | transgenic confirmation | XbaI intron-insert | CGATAAGCTTGGATCCTCTAG | AttB1ABC | GGGGACAAGTTTGTACAAAAAAGCAGGCTTGA  TTGATGGTGAAGGCAAG | 55oC | none | 400+113 |
| pSP13-1 | transgenic confirmation | XhoI intron-insert | GCTGCAGCTGGATGGCAAAT | AttB1ABC | GGGGACAAGTTTGTACAAAAAAGCAGGCTTGA  TTGATGGTGAAGGCAAG | 55oC | none | 403+117 |
| S1 | INDEL | T16305F | AAACTCAAACATTAGAAAGACTTGAAA | 3utrR | GAGTTATAAAGAAACAAGATCCGTTC | 60oC | none | 400 |
| S106_1 | INDEL | S106-indel1F | CGGTGGAGTCCCTGATAAAT | S106-indel1R | TGTTATATAAATTTCAAAGTTGAGTGACC | 60oC | none | 370/400 |
| S106_2 | INDEL | S106-indel2F | CAAAAGGTCACTCAACTTTGAAAT | S106-indel2R | CAAATGAACAGCCACAATCG | 60oC | none | 370/400 |
| S108 | CAPS | S108F | CTGGGAATTGGAAAGAGTCG | S108R | TGCAAACCTCACAAGGATCA | 60oC | AflII | 650/870 |
| S16500 | CAPS | S16500F | GAGTCTTTTGGGATGCTGGA | S16500R | CAAATACATGTCCGGGGAGT | 60oC | NlaIII | 410/500 |
| S22500 | CAPS | S22500F | GAAATGGCTCTGATTCCCATA | S22500R | TGTTGGAGACTTCCGCTTCT | 60oC | EcoRV | 950/750 |
| S27 | CAPS | S27F | ATTAGGAGCTCTATTATCATGTCAACT | S27R | CAAGATTTTGTCCCACCTAATTT | 60oC | TaqI | 800/700 |
| S38 | CAPS | S38F | GCACTCACAGTAACCCTTGC | S38R | TGGGTGGTTTTCTTTTTAACTTG | 60oC | XhoI | 400/800 |
| S41 | CAPS | S41F | AAATTGACAAAGTAAAGTCAAGATTCA | S41R | TCCAACAGGAAACAACAATCA | 60oC | TaqI | 500/400 |
| S47 | CAPS | S47F | GCCCAAATATGGCATAAACAA | S47R | TGAGTACTGAATTATGAGCGTCAA | 60oC | TaqI | 150/350 |
| S85 | CAPS | S85F | TTGCTGAAAAGTTGGAGAGTGA | S85R | CCAAAGAGATAGACCATTACACGA | 60oC | RsaI | 850/800 |
| S87 | CAPS | S87F | TTCTCTAAGGCTAGAACATGTAAAAA | S87R | TGTTCTTGGTGTTTGCGAAG | 60oC | EcoRI | 850/1000 |
| S91 | CAPS | S91F | CCTGTAGAGGTAGGCGGAAA | S91R | TCCCCTTTTTGGTTTTGTTAAA | 60oC | TaqI | 400/500 |
| S93 | CAPS | S93F | TTCCTACCCCCGTAAAAGAA | S93R | TGGAAAATTAAAGAGCACCACA | 60oC | HinfI | 500/400 |
| S98 | CAPS | S98F | CGCTGAACTTTCATGTGTAGCA | S98R | GGGAGTAATTGTCAATGTTTCG | 60oC | HinfI | 600/850 |
| SS1 | CAPS | SS1F | GACTGAACTTAAAATCCTATACAGACC | SS1R | AAGATTGTATTTATGGTGTCATGC | 60oC | TaqI | 756/450 |
| SS2 | CAPS | SS2F | TGGTATTTCAACTCCTCTTCATACC | SS2R | TCTGCACTTCTGAGCAAGATTC | 60oC | HaeIII | 744 |
| SS4 | CAPS | SS4F | GTTTTCACCTGATTTCACTATTGTT | SS4R | GAGACAAGGCACAAGGGAAG | 60oC | SspI | 800/500 |
| SS7 | dCAPS | SS7F | TTGACCGAACCACGTTAAATC | dss7Reco | CAGTTTTGTCCAATTTTTTGCTCGAATGAATT | 60oC | EcoRI | 600 |
| SSR1S2 | SSR | SSR1F2 | CGATTAAGGGCAAATATTAATTTACTT | SSR1R2 | TTCTCTTAATGTGTTTGTGTCACG | 60oC | none | 400/370 |
| SSR2S2 | SSR | SSR2F2 | GGCATTTGACAATGTAATTTGG | SSR2R2 | TTTACAAGCATCCGTTATTCCA | 60oC | none | 300/270 |
| ST4 | CAPS | ST4F | GCTTCAAAATGATATTTCAATAGTGT | ST4R | TGGAATCAAAAGGGTGTAGTGA | 60oC | AluI | 651/450 |
| T1317 | CAPS | T1317F | GCTCAACGTTCAATCTTCATAGC | T1317R | CTGTTTTCAAACAAATCACCACA | 60oC | EcoRV | 2100 |
| T1322 | dCAPS | T1322F | CGTTGTTGGTGCTACAATTAAA | dT1322 | GAGTCTATCAGTTCAAATAAAAAAAAAGTTCG | 60oC | TaqI | 200 |
| T1630 | INDEL | T1630F1 | CTTTTAAAGGATGGGGCACA | T1630R1 | GAAGGCGAAAACAAAACACAA | 60oC | NO | 2100/2250 |
| T635 | CAPS | T635F2 | TCAACCAACAACAAGGGTCA | T635R2 | CCAGGAGCATCACAGTCAAA | 60oC | Alu I | 900 |
| T725 | dCAPS | dCT97F2 | TGGGGGTGAGGCGGAGGGAGTGAAG | dCT97R3 | TTGAAACAACCTGACAGGAG | 53oC | AluI | 370/400 |
| T872 | CAPS | T872AF | CCGGAGTAGGAACAAGTTGG | T872AR | CTGTCTCCCGATTCATTCGT | 53oC | MseI | 1000 |
| T877 | dCAPS | DCAPS-SAC-F | CAAGAAATCGACAAAGCGAGTGAG | DCAPS-SAC-R | TCATCCCTTATCGAGGCTTG | 53oC | SacI | 450 |
| T954 | dCAPS | T954F | ATGGCAAATTTGTTCATTAAGC | dT954R | CGATCAATGAACACTCAAATTATAG | 60oC | EcoRI | 350 |
| TG2 | dCAPS | TG2F | CCTCAATCCCAGGAATTTGT | dTG2R | GCTAAATAGGAGCTTTGTTTAAATTTTGCG | 60oC | RsaI | 250 |
| TG516 | dCAPS | TG516F | TGCCTGCAGTACATGTGAAGA | dTG516R | CCTGCTTTAATCTCCAATCTTTTCTCTGATTC | 60oC | TaqI | 350 |
| TG65 | CAPS | TG65F | CTTGCACAGAATGCTCCTTG | TG65R | GGGGGACGTACATGAAGCTA | 60oC | HhaI | 1550/1400 |
| U43768 | CAPS | U43768F | GAATGGGTTGTTCTCGGCTA | U43768R | CATTGAGCAATTCACAAGTCG | 53oC | Hinf I | 500 |
| U49812 | CAPS | U49812F | CAGGAAAAGCAAAGCCAAAG | U49812R | TCAAGCTGAGCATGCTTTTT | 53oC | EcoRV | 500 |
